# Clinical significance of CD133 and Nestin in astrocytic tumor: The correlation with pathological grade and survival

**DOI:** 10.1002/jcla.23082

**Published:** 2019-11-01

**Authors:** Qingping Zhang, Binchu Xu, Jianliang Chen, Furong Chen, Zhongping Chen

**Affiliations:** ^1^ Department of Neurosurgery Huazhong University of Science and Technology Union Shenzhen Hospital Shenzhen China; ^2^ Department of Neurosurgery The Seventh Affiliated Hospital of Sun Yat‐sen University Shenzhen China; ^3^ Department of Neurosurgery The Eighth Affiliated Hospital Sun Yat‐sen University Shenzhen China; ^4^ Department of Neurosurgery/Neuro‐oncology Sun Yat‐sen University Cancer Center Guangzhou China; ^5^ State Key Laborarory of Oncology in South China Guangzhou China

**Keywords:** astrocytic tumor, CD133, Nestin, overall survival, World Health Organization grade

## Abstract

**Background:**

We aimed to investigate the interaction between CD133 and Nestin and further assessed the correlation of CD133 and Nestin with clinicopathological characteristics and survival in patients with astrocytic tumor.

**Methods:**

Totally 127 patients with astrocytic tumor underwent surgical resection were enrolled. Patients’ age, gender, and World Health Organization (WHO) grade were recorded, and the survival data were extracted from the follow‐up records. The expressions of CD133 and Nestin in astrocytic tumor tissues were analyzed by immunohistochemistry assay. The WHO grade I and II astrocytic tumors were defined as low‐grade astrocytic tumors (LGA), the WHO grade III and IV astrocytic tumors were defined as high‐grade astrocytic tumors (HGA).

**Results:**

There were 79 (62.2%), 34 (26.8%), 14 (11.0%), and 0 (0.0%) patients with CD133 negative, low, moderate, and high expression, respectively; 7 (5.5%), 47 (37.0%), 20 (15.7%), 53 (41.7%) patients with Nestin negative, low, moderate, high expression, respectively. CD133 and Nestin were both correlated with advanced WHO grade but not with age or gender, and positive correlation was observed between CD133 and Nestin. For survival, both CD133 and Nestin were correlated with unfavorable overall survival (OS), and further analysis illustrated that Nestin but not CD133 independently predicted poor OS. Subgroup analysis also revealed that Nestin but not CD133 negatively associated with shorter OS in LGA patients, while both CD133 and Nestin were correlated with poor OS in HGA patients.

**Conclusion:**

CD133 and Nestin present as potential biomarkers for advanced pathological grade and poor survival in patients with astrocytic tumor.

## 1 INTRODUCTION

Astrocytic tumor is the most common glial tumor of the central nervous system, accounting for 13%‐26% of intracranial tumors.^1^ It is characterized by fast and invasive growth as well as no obvious boundary with normal brain tissue, which leads to short course of the disease and poor prognosis.^2^ Currently, the treatment for astrocytic tumor relies on surgery, radiotherapy and chemotherapy.^1^ Surgical resection is theoretically a cure for astrocytic tumor; however, due to the infiltration and frequent recurrence of the tumor, surgical resection is incomplete and ineffective in a proportion of cases, or even hard to be carried out when tumor grows in certain important areas such as brain stem.^3^ Radiotherapy is the common therapy for almost all types of glial tumors including astrocytic tumor. However, only a few subtypes present high sensitivity to radiotherapy and the effect of radiation‐induced necrosis on brain function should not be underestimated.^4^, ^5^, ^6^ In addition, chemotherapy is limited to blood‐brain barrier and side effects. Therefore, it is of clinical significance to explore potential biomarkers for disease monitoring and prognosis prediction, which would assist with treatment of astrocytic tumor.

In recent years, researchers discover the existence of cancer stem cells that have self‐renewal and immense differentiation ability in astrocytic tumor. These cells are insensible and highly resistant to current radiotherapy and chemotherapy and contribute to the tumor metastasis and recurrence.^7^, ^8^ Identification of cancer stem cells has been realized via a number of markers, and undoubtedly, the cancer stem cell markers provide a new direction for the treatment of astrocytic tumor and the prevention of recurrence.^9^ CD133, a brain tumor stem cell marker, is shown to be upregulated in recurrent pilocytic astrocytoma, and suppression of CD133 increases cell chemosensitivity via inhibiting PI3K‐Akt‐NF‐κB signaling mediators and MDR1.^10^ In addition, the intermediate filament protein Nestin, which is critical in central nervous system development, is correlated with higher WHO grade and poor prognosis in patients with astrocytic tumor.^11^, ^12^ However, current studies investigating the correlation of CD133 and Nestin with clinicopathological features and prognosis are mostly based on general glioma patients or astrocytic tumor patients in a specific pathological stage. Therefore, in this study, we evaluated the interaction between CD133 and Nestin and further assessed the correlation of CD133 and Nestin with clinicopathological characteristics and survival in patients with astrocytic tumor.

## 2 MATERIALS AND METHODS

### 2.1 Patients and tumor specimens

A total of 127 patients with astrocytic tumor who underwent surgical resection at our hospital between January 2005 and December 2011 were reviewed and analyzed in this study. All reviewed patients had (a) newly diagnosed as primary astrocytic tumor by pathological examination, (b) sufficient available tumor tissue for immunohistochemical analysis, (c) detailed clinical and follow‐up data, and (d) without neoadjuvant therapy before surgery. All 127 human astrocytic tumor specimens were removed from surgical resection, confirmed by the pathology, fixed in the 10% neutral formaldehyde and embedded in paraffin wax. This study was approved by the Institutional Review Board of Shenzhen Nanshan People's Hospital. Written informed consents or verbal agreements with tape recording were collected from reviewed patients or their guardians.

### 2.2 Data collection

The clinical data of studied cases were collected from the medical records, including age, gender and World Health Organization (WHO) grade. The assessment of WHO grade was based on the 2000 WHO classification of nervous system tumors,^13^ and the WHO grade I and II astrocytic tumors were defined as low‐grade astrocytic tumors (LGA), the WHO grade III and IV astrocytic tumors were defined as high‐grade astrocytic tumors (HGA). The postoperative treatments including chemotherapy, radiotherapy (50‐60Gy/5W), and immunotherapy were administered to patients based on the clinical status. All patients were followed up by telephone or clinic visit, with a median follow‐up of 24.0 months (ranging from 2 to 113 months), and the survival data were extracted from the follow‐up records, which were used to calculate the overall survival (OS).

### 2.3 Immunohistochemistry (IHC) assay

The expression of CD133 and Nestin in astrocytic tumor tissue was analyzed by IHC staining, which was performed using the Rabbit anti‐human CD133 monoclonal antibody (Cell Signaling Technology, Inc) and the Mouse anti‐human Nestin monoclonal antibody (Santa Cruz Biotechnology, Inc) served as primary antibody, respectively. IHC assay was carried out as follows: (a) formalin‐fixed paraffin‐embedded astrocytic tumor tissues were cut into 4 µm sections, then were baked in oven at 60℃ for 30 minutes; (b) deparaffinizing in xylene and rehydration in a graded series of ethanol were preformed; (c) for antigen retrieval, the sections were placed in 0.01 mol/L citrate buffer (pH 6.0) and boiled for 20 minutes; (d) to block endogenous peroxidase, each section was incubated at room temperature with 3% H_2_O_2_ for 10 minutes; (e) for immunoreaction, the section was incubated with primary antibody (Rabbit anti‐human CD133 monoclonal antibody or Mouse anti‐human Nestin monoclonal antibody) at 37℃ for 2 hours (negative control was incubated with phosphate buffer saline (PBS) instead of primary antibody); (f) after incubated with the primary antibody, the section was incubated with horseradish peroxidase‐conjugated goat‐anti‐rabbit/goat‐anti‐mouse immunoglobulin G antibody (Santa Cruz Biotechnology, Inc); (g) newly prepared diaminobenzidine (DAB) solution were added to each section for visualization; (h) the section was washed using distilled water, followed by the counterstaining with hematoxylin for 1 minute; and (i) after routine dehydration, transparency, drying and sealing, the section was observed on the microscope.

### 2.4 IHC assessment

CD133 was a transmembrane protein, located in the cell membrane and cytoplasm. The positive staining cells showed immunoreactivity on the cell membranes and cytoplasm, presenting with brown‐yellow brighter than background color. The percentage of positive cells in ten randomly selected fields under 200× magnification was calculated. Nestin was located in the cytoplasm, and the positive staining cells was presented as reddish‐brown granules in the cytoplasm. Under 200× magnification, the percentage of positive cells in ten randomly selected fields was calculated as well. Both CD133 and Nestin staining were semi‐quantitatively graded for the percentage of positive tumor cells as the method that previous studies report 14, 15: negative (0%): no tumor cell was positively stained; low (<30%): <30% of the tumor cells were positively stained; moderate (30%‐60%): 30%‐60% of the tumor cells were positively stained; high (≥60%): ≥60% of the tumor cells were positively stained.

### 2.5 Statistical analysis

Data were presented as count (percentage). Correlation analysis was performed using the Chi‐square test or Spearman's rank correlation test. OS was calculated from primary surgery until death or date of censoring, and the OS was illustrated using the Kaplan‐Meier curves and compared by the log‐rank test. Factors predicting OS were assessed by the use of univariable and multivariable Cox's proportional hazard regression model analyses. A *P* value < .05 was considered significant. All data analysis was performed using the SPSS 22.0 statistical software (IBM), and the graph was made using the GraphPad Prism 7.02 software (GraphPad Software Inc).

## 3 RESULTS

### 3.1 Correlation of CD133 and Nestin with clinical features

**Figure 1:**
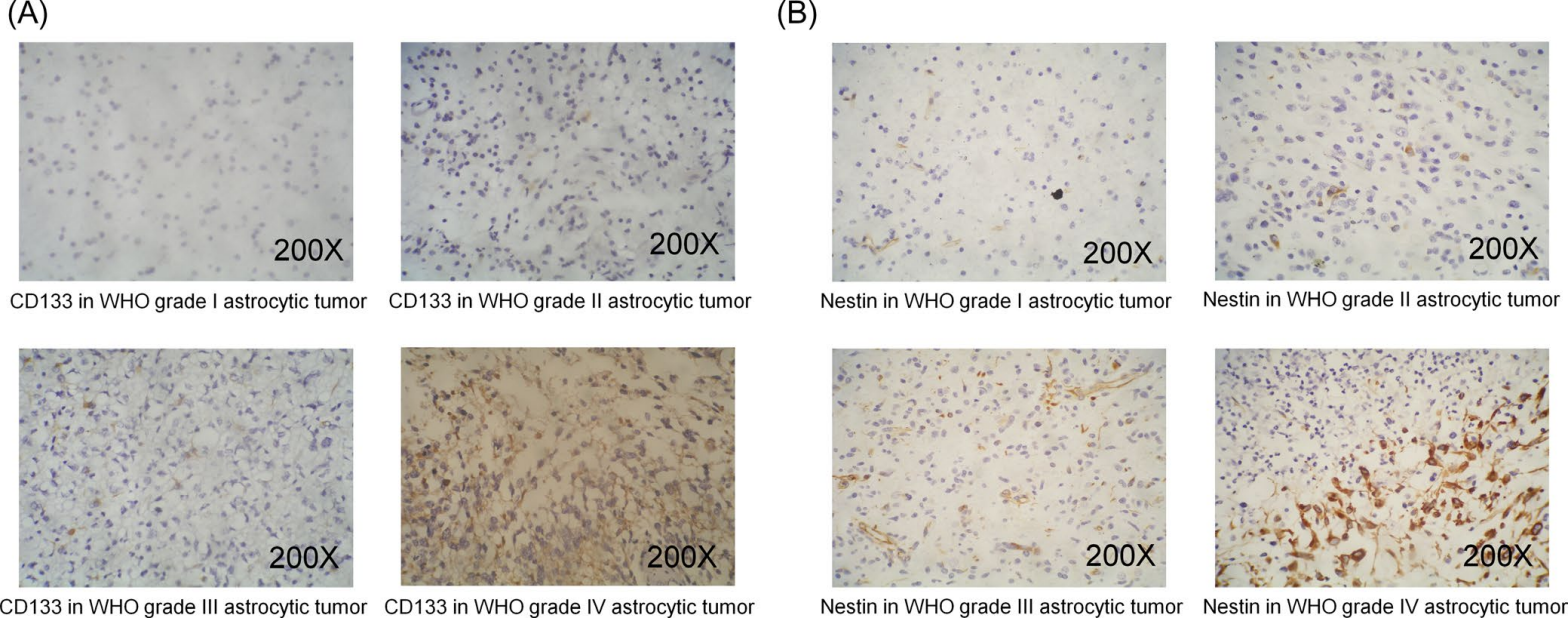
Immunohistochemistry (IHC) staining for expression of CD133 and Nestin in astrocytic tumors. The expression of CD133 (A) and Nestin (B) in astrocytic tumor at different WHO grades was shown. IHC, immunohistochemistry; WHO, World Health Organization

There were 79 (62.2%), 34 (26.8%), 14 (11.0%), and 0 (0.0%) patients with CD133 negative, low, moderate, and high expression, respectively. CD133 expression was correlated with advanced WHO grade (*P* < .001) (Table 1, Figure 1A). However, no correlation was observed between CD133 and age (*P* = .566) or gender (*P* = .115). As for Nestin, 7 (5.5%), 47 (37.0%), 20 (15.7%), and 53 (41.7%) patients were with Nestin negative, low, moderate, and high expression, respectively. Nestin was associated with advanced WHO grade (*P* < .001) (Table 1, Figure 1B). However, no correlation was observed between Nestin and age (*P* = .628), gender (*P* = .056).

**Table 1:** Correlation of CD133 and Nestin expressions with clinical characteristics

Items	Total patients	CD133 expression					Nestin expression				
		Negative (0%)	Low (0%‐30%)	Moderate 30%‐60%	High (≥60%)	*P* value	Negative (0%)	Low (0%‐30%)	Moderate (30%‐60%)	High (≥60%)	*P* value
No. of patients	127	79	34	14	0		7	47	20	53	
Age, No. (%)											
≤40 y	80 (63.0)	51 (63.7)	22 (27.5)	7 (8.8)	0 (0.0)	.566^a^	5 (6.2)	31 (38.8)	14 (17.5)	30 (37.5)	.628^a^
>40 y	47 (37.0)	28 (59.6)	12 (25.5)	7 (14.9)	0 (0.0)		2 (4.3)	16 (34.0)	6 (12.8)	23 (48.9)	
Gender, No. (%)											
Male	75 (59.1)	48 (64.0)	16 (21.3)	11 (14.7)	0 (0.0)	.115^a^	2 (2.7)	29 (38.6)	8 (10.7)	36 (48.0)	.056^a^
Female	52 (40.9)	31 (59.6)	18 (34.6)	3 (5.8)	0 (0.0)		5 (9.6)	18 (34.6)	12 (23.1)	17 (32.7)	
WHO grade, No. (%)											
I	15 (11.8)	13 (86.7)	2 (13.3)	0 (0.0)	0 (0.0)	<.001^b^	7 (46.6)	4 (26.7)	3 (20.0)	1 (6.7)	<.001^b^
II	42 (33.1)	31 (73.8)	11 (26.2)	0 (0.0)	0 (0.0)		0 (0.0)	22 (52.4)	7 (16.7)	13 (30.9)	
III	42 (33.1)	28 (66.7)	9 (21.4)	5 (11.9)	0 (0.0)		0 (0.0)	18 (42.9)	4 (9.5)	20 (47.6)	
IV	28 (22.0)	7 (25.0)	12 (42.9)	9 (32.1)	0 (0.0)		0 (0.0)	3 (10.7)	6 (21.4)	19 (67.9)	

Abbreviations: SD, standard deviation; WHO, World Health Organization.

a Chi‐square test.

b Spearman's rank correlation test.

### 3.2 Correlation of CD133 with Nestin

In order to determine the interaction between CD133 and Nestin, Spearman's rank correlation test was performed, which observed that CD133 expression was positively correlated with Nestin expression in astrocytic tumor tissue (*P* < .001, *r* = .299) (Table 2).

**Table 2:** Correlation of CD133 expression with Nestin expression

Items		CD133 expression				*P* value	*r*
		Negative (0%)	Low (0%‐30%)	Moderate (30%‐60%)	High (≥60%)		
	No.	79	34	14	0		
Nestin expression							
Negative (0%)	7	7 (5.5)	0 (0.0)	0 (0.0)	0 (0.0)	<.001	.299
Low (0%‐30%)	47	35 (27.6)	11 (8.7)	1 (0.8)	0 (0.0)		
Moderate (30%‐60%)	20	9 (7.1)	11 (8.7)	0 (0.0)	0 (0.0)		
High (≥60%)	53	28 (22.0)	12 (9.4)	13 (10.2)	0 (0.0)		

Note

Correlation was determined by Spearman's rank correlation test. *r*, correlation coefficient.

### 3.3 Correlation of CD133 and Nestin with OS

**Figure 2:**
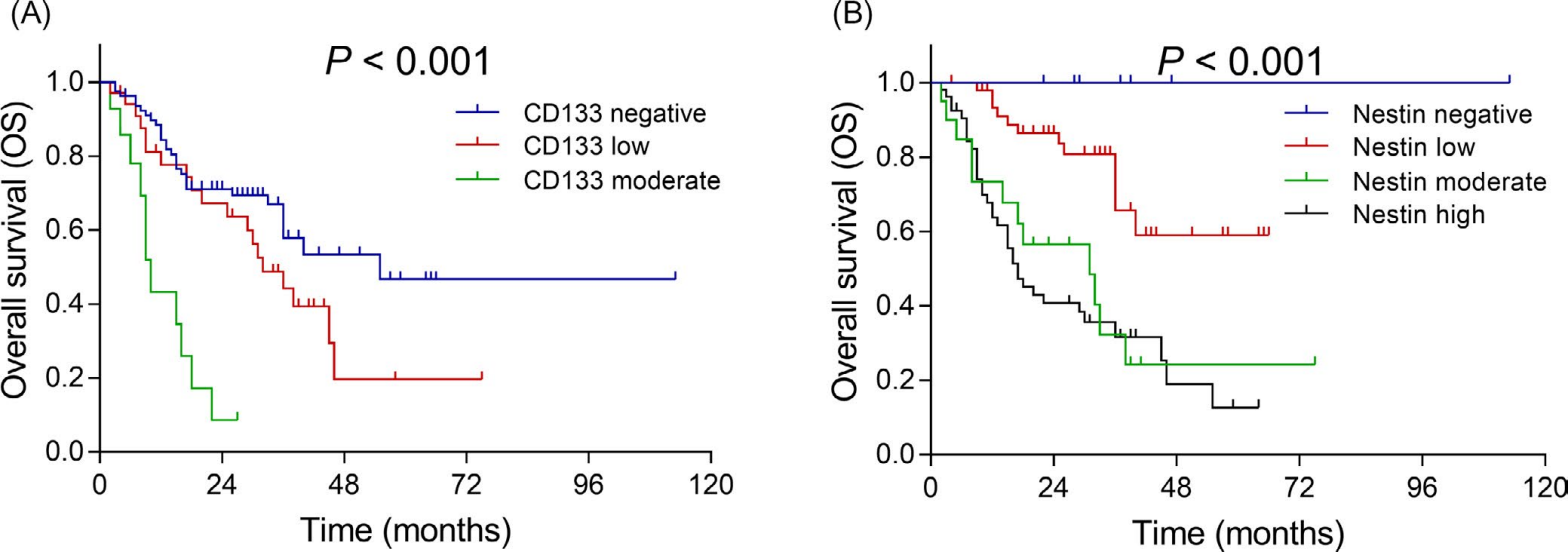
Comparison of OS among patients with different CD133 and Nestin expressions. The correlation of CD133 (A) and Nestin (B) with OS in patients with astrocytic tumor. OS was illustrated using the Kaplan‐Meier curves and compared by the log‐rank test. *P* < .05 was considered significant. OS, overall survival

Patients with CD133 moderate expression presented with the lowest OS, followed by patients with CD133 low expression, and patients with CD133 negative, further analysis illustrated that CD133 was negatively correlated with OS (*P* < .001) (Figure 2A). Regarding Nestin, patients with Nestin high expression showed the lowest OS, followed by patients with Nestin moderate expression, patients with Nestin low expression, and then patients with Nestin negative, the subsequent analysis indicated that Nestin was negatively correlated with OS (*P* < .001) (Figure 2B).

### 3.4 Factors affecting OS

Univariate Cox's proportional hazard model regression disclosed that higher CD133 expression (*P* < .001, HR = 2.186), higher Nestin expression (*P* < .001, HR = 2.033), age (>40 vs ≤40 years) (*P* = .001, HR = 2.401), gender (male vs female) (*P* = .005, HR = 2.238), and higher WHO grade (*P* < .001, HR = 2.852) predicted poor OS, while radiotherapy predicted longer OS (*P* = .029, HR = 0.538) (Table 3). And multivariate Cox's proportional hazard model regression further showed that higher CD133 expression (*P* = .640, HR = 0.895), radiotherapy (*P* = .124, HR = 0.569) were not independent predictive factors for OS, whereas higher Nestin expression (*P* = .004, HR = 1.611), age (>40 vs ≤40 years) (*P* = .003, HR = 2.319), gender (male vs female) (*P* = .006, HR = 2.457), and higher WHO grade (*P* < .001, HR = 2.607) independently predicted poor OS.

**Table 3:** Univariate and multivariate Cox's proportional hazard model regression analyses of factors affecting OS

Items	Univariate Cox's regression		Multivariate Cox's regression	
	*P* value	HR (95%CI)	*P* value	HR (95%CI)
Higher CD133 expression^a^	<.001	2.186 (1.515‐3.155)	.640	0.895 (0.561‐1.426)
Higher Nestin expression^a^	<.001	2.033 (1.520‐2.719)	.004	1.611 (1.166‐2.227)
Age (>40 vs ≤40 y)	.001	2.401 (1.435‐4.016)	.003	2.319 (1.331‐4.041)
Gender (male vs female)	.005	2.238 (1.268‐3.951)	.006	2.457 (1.298‐4.649)
Higher WHO grade	<.001	2.852 (2.049‐3.969)	<.001	2.607 (1.759‐3.864)
Radiotherapy (yes vs no)	.029	0.538 (0.309‐0.938)	.124	0.569 (0.277‐1.166)
Chemotherapy (yes vs no)	.248	0.723 (0.417‐1.253)	.955	0.982 (0.516‐1.869)
Immunotherapy (yes vs no)	.121	0.446 (0.161‐1.238)	.070	0.332 (0.101‐1.094)

Abbreviations: CI, confidence; HR, hazard ratio; OS, overall survival; WHO, World Health Organization.

a CD133 and Nestin expression in the Cox's regression analysis in the form of ordered categorical variable, which were encoded as: negative (0%) = 0, low (0%‐30%) = 1, moderate (30%‐60%) = 2, high (≥60%) = 3; WHO grade in the Cox's regression was encoded as: grade I = 1, grade II = 2, grade III = 3, grade IV = 4.

### 3.5 Correlation of CD133 and Nestin with OS in LGA patients and HGA patients

**Figure 3:**
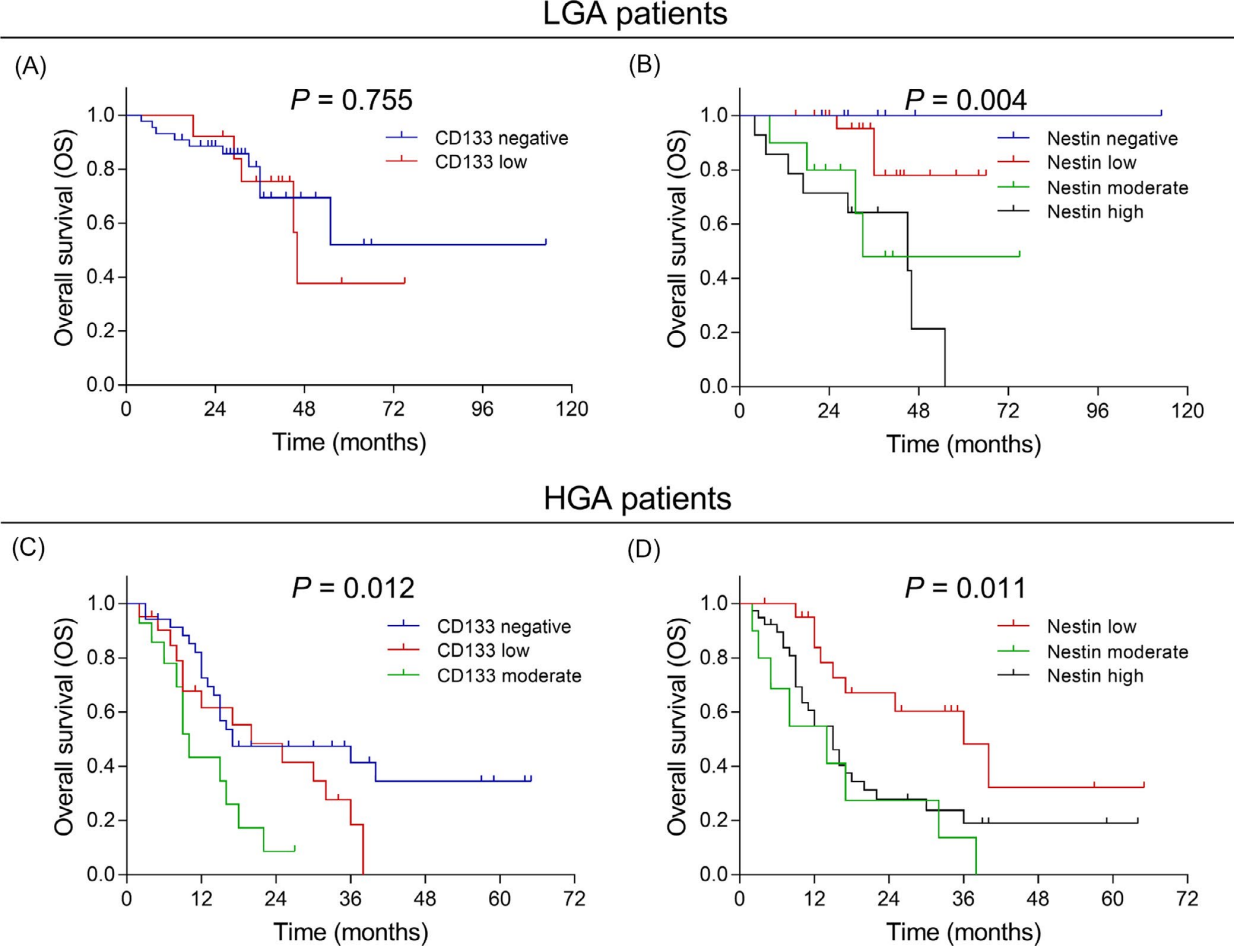
Subgroup analysis for comparison of OS among patients with different CD133 and Nestin expressions. The correlation of CD133 (A) and Nestin (B) with OS in LGA patients. The correlation of CD133 (C) and Nestin (D) with OS in HGA patients. OS was illustrated using the Kaplan‐Meier curves and compared by the log‐rank test. *P* < .05 was considered significant. OS, overall survival; LGA, low‐grade astrocytic tumors; HGA, high‐grade astrocytic tumors

The correlation of CD133 and Nestin with OS were then evaluated separately in LGA patients and HGA patients. In LGA patients, CD133 was not correlated with OS (*P* = .775) (Figure 3A), whereas Nestin was negatively correlated with OS (*P* = .004) (Figure 3B). In HGA patients, both CD133 (*P* = .012) (Figure 3C) and Nestin (*P* = .011) (Figure 3D) were negatively associated with OS.

### 3.6 Factors affecting OS in LGA patients and HGA patients

Univariate Cox's regression showed that higher Nestin expression (*P* = .001, HR = 2.551) and age (>40 vs ≤40 years) (*P* = .030, HR = 3.155) were correlated with worse OS, while higher CD133 expression (*P* = .756, HR = 1.187), gender (male vs female) (*P* = .084, HR = 2.663), radiotherapy (*P* = .931, HR = 1.049), chemotherapy (*P* = .937, HR = 1.042), and immunotherapy (*P* = .681, HR = 0.652) were not correlated with OS in LGA patients (Table 4). Multivariate Cox's regression further exhibited that higher Nestin expression (*P* = .008, HR = 2.342) and age (>40 vs ≤40 years) (*P* = .009, HR = 5.979) independently predicted shorter OS, whereas higher CD133 expression (*P* = .860, HR = 1.144), gender (male vs female) (*P* = .096, HR = 3.316), radiotherapy (*P* = .943, HR = 1.059), chemotherapy (*P* = .624, HR = 0.701), and immunotherapy (*P* = .936, HR = 1.105) were not independent predictive factors for OS in LGA patients (Table 4). As for HGA patients, univariate Cox's regression showed that higher CD133 expression (*P* = .005, HR = 1.731) and higher Nestin expression (*P* = .037, HR = 1.440) were correlated with shorter OS, while radiotherapy (*P* = .018, HR = 0.445) and immunotherapy (*P* = .026, HR = 0.254) were correlated with longer OS, whereas age (>40 vs ≤40 years) (*P* = .259, HR = 1.409) and gender (male vs female) (*P* = .089, HR = 1.785) were not predictive factors for OS in HGA patients (Table 5). Multivariate Cox's regression further illustrated that higher Nestin expression independently predicted poor OS in HGA patients (*P* = .023, HR = 1.584) and immunotherapy independently predicted longer OS (*P* = .042, HR = 0.214), while higher CD133 expression (*P* = .625, HR = 1.131), age (>40 vs ≤40 years) (*P* = .162, HR = 1.533), gender (*P* = .110, HR = 1.846), radiotherapy (*P* = .306, HR = 0.637), and chemotherapy (*P* = .376, HR = 1.436) were not independent predictive factors for OS in HGA patients (Table 5).

**Table 4:** Univariate and multivariate Cox's proportional hazard model regression analyses of factors affecting OS in LGA patients

Items	Univariate Cox's regression		Multivariate Cox's regression	
	*P* value	HR (95%CI)	*P* value	HR (95%CI)
Higher CD133 expression^a^	.756	1.187 (0.402‐3.502)	.860	1.144 (0.255‐5.142)
Higher Nestin expression^a^	.001	2.551 (1.444‐4.507)	.008	2.342 (1.248‐4.396)
Age (>40 vs ≤40 y)	.030	3.155 (1.117‐8.912)	.009	5.979 (1.572‐22.748)
Gender (male vs female)	.084	2.663 (0.877‐8.089)	.096	3.316 (0.809‐13.585)
Radiotherapy (yes vs no)	.931	1.049 (0.358‐3.077)	.943	1.059 (0.218‐5.136)
Chemotherapy (yes vs no)	.937	1.042 (0.374‐2.908)	.624	0.701 (0.169‐2.903)
Immunotherapy (yes vs no)	.681	0.652 (0.085‐4.993)	.936	1.105 (0.095‐12.904)

Abbreviations: CI, confidence; HR, hazard ratio; LGA, low‐grade astrocytic tumor (WHO grade I ~ II);

OS, overall survival; WHO, World Health Organization.

a CD133 and Nestin expression were included in the Cox's regression in the form of ordered categorical variable, which were encoded as: negative (0%) = 0, low (0%‐30%) = 1, moderate (30%‐60%) = 2, high (≥60%) = 3.

**Table 5:** Univariate and multivariate Cox's proportional hazard model regression analyses of factors affecting OS in HGA patients

Items	Univariate Cox's regression		Multivariate Cox's regression	
	*P* value	HR (95%CI)	*P* value	HR (95%CI)
Higher CD133 expression^a^	.005	1.731 (1.175‐2.549)	.625	1.131 (0.690‐1.853)
Higher Nestin expression^a^	.037	1.440 (1.021‐2.031)	.023	1.584 (1.067‐2.351)
Age (>40 vs ≤40 y)	.259	1.409 (0.777‐2.554)	.162	1.553 (0.838‐2.879)
Gender (male vs female)	.089	1.785 (0.916‐3.478)	.110	1.846 (0.871‐3.915)
Radiotherapy (yes vs no)	.018	0.445 (0.228‐0.871)	.306	0.637 (0.269‐1.510)
Chemotherapy (yes vs no)	.414	0.755 (0.384‐1.484)	.376	1.436 (0.644‐3.202)
Immunotherapy (yes vs no)	.026	0.254 (0.076‐0.848)	.042	0.214 (0.049‐0.943)

Abbreviations: CI, confidence; HGA, high‐grade astrocytic tumor (WHO grade III ~ IV); HR, hazard ratio; OS, overall survival; WHO, World Health Organization.

a CD133 and Nestin expression were included in the Cox's regression in the form of ordered categorical variable, which were encoded as: negative (0%) = 0, low (0%‐30%) = 1, moderate (30%‐60%) = 2, high (≥60%) = 3.

## 4 DISCUSSION

There are a small population of highly tumorigenic cancer stem cells existed in a variety of malignancies including astrocytic tumor.^7^ The presence of cancer stem cells drives the invasiveness of tumor and resistance to therapies, which is crucial for tumor metastasis and relapse.^8^The isolation of cancer stem cells is achieved via specific stem cell‐related surface antigens, and the abundance of these cancer stem cell markers are shown to be closely related with cancer development and progression.^9^ For instance, the transmembrane glycoprotein CD133 has been used to identify cancer stem cells in different solid tumors including brain, lung, gastric, and liver cancers.^16^ In brain tumors, CD133 is first used to identify cancer stem cells in pediatric samples of glioma and medulloblastoma, and CD133 positive tumor cells are more aggressive and with higher capacity to self‐renew.^9^, ^17^ And in astrocytic tumor, CD133 expression is correlated with higher histological grade and larger tumor size.^18^ In addition, Nestin is one of the type VI intermediate filament proteins. It regulates the stemness of different cancer cells and promotes tumor invasion and metastasis.^14^ In astrocytic tumors, Nestin is abundantly expressed in undifferentiated stem cells and progenitors cells, and it is associated with increased WHO grade, but not with patient's gender, age, tumor location or tumor size.^19^ Besides, Nestin positive glioblastoma cells show increased tumor sphere‐forming ability and tumor sphere size and is correlated with high grades of malignancy in glioblastoma.^20^ However, the patients enrolled in these previous studies were mostly general glioma patients or with certain histological subtype of astrocytic tumors, and the sample sizes were relatively small. Thus, we recruited a larger number of patients with astrocytic tumor, and our study reported that CD133 and Nestin expressions were both correlated with advanced WHO grade but not with age or gender in patients with astrocytic tumor. In addition, positive correlation was also observed between CD133 and Nestin. The following are the possible explanations. (a) High CD133 and Nestin levels represent the abundance of cancer stem cells or stem cell‐like cells, which are capable of splicing symmetrically and asymmetrically into tumor precursor cells and contribute to heterogeneity of tumor. Therefore, CD133 and Nestin high expression forecasts higher WHO grade. (b) Both CD133 and Nestin are indicators for stemness and are increasingly expressed in tumors with higher WHO grade as shown by IHC staining; the elevation of any one of them indicates enhanced stemness, which in turn correlates with the increased expression of another one.

CD133 and Nestin have also been studied regarding their impact on prognosis in a range of solid tumors. For instances, overexpression of CD133 confers poor prognosis in invasive breast cancer, colorectal cancer, pancreatic cancer, etc.^21^, ^22^, ^23^ In addition, the negative impact of Nestin on survival has also been observed in lung cancer, esophageal squamous cancer, bladder cancer, etc.^24^, ^25^, ^26^ Whereas in astrocytic tumor, the tumor of the central nervous system, the effects of CD133 and Nestin on prognosis are only discovered in a small volume of literatures.^18^, ^19^ Therefore, this present study recruited relatively more patients with astrocytic tumor compared with the previous literatures and aimed to further validate the clinical relevance of CD133 and Nestin in astrocytic tumor regarding their impact on survival. We disclosed that CD133 and Nestin both predicted poor OS in patients with astrocytic tumor. The possible reasons are as follows: (a) CD133 high expression is a sign of cancer stem cell activity that increases cell differentiation and proliferation, which contributes to tumor progression; Nestin is previously shown to delineate invasion of astrocytic tumor into the adjacent gray and white matter; therefore, Nestin is associated with aggressive tumor behavior, which leads to higher pathological grade and poor prognosis in patients with astrocytic tumor.^14^, ^16^ (b) Increased stemness is also shown to induce resistance to chemotherapy and radiotherapy, which leads to poor prognosis in patients with astrocytic tumor.^10^

In subgroup analysis, CD133 was correlated with poor OS in HGA patients but not in LGA patients. This may be due to that LGA tumors present extremely low level of CD133 as shown by IHC staining, and the number of LGA patients was relatively small, which leads to reduced statistical power; therefore, it is possible that the correlation of CD133 with OS is not observed in LGA patients. Additionally, although Nestin was correlated with shorter OS in both LGA and HGA patients, it was not an independent predictive factor for OS in HGA patients. This may be account for that in HGA patients, other characteristics such as higher CD133 expression and high WHO grade are cofounding factors influencing OS, thus, Nestin is not observed to be an independent risk factor.

Although CD133 and Nestin were shown to be correlated with advanced WHO grade and poor survival in our study, the detailed mechanisms of CD133 and Nestin in regulating cell stemness and tumor progression in astrocytic tumor were not investigated. Besides, a large scaled study with greater sample size is still needed to further validate our findings. Additionally, due to relatively early patient enrollment time, WHO classification 2000 was used for tumor evaluation, which was a bit old compared with the newly launched WHO classification 2016. However, due to lack of cytogenic and molecular genetics information, re‐evaluation of the tumors using the 2016 classification was not feasible. And the correlation of IDH mutation with CD133 and Nestin as well as survival was not assessed.

In conclusion, CD133 and Nestin are correlated with advanced WHO grade and poor survival in patients with astrocytic tumor, which benefits the exploration of prognostic factor for astrocytic tumor.

## CONFLICTS OF INTEREST

The authors declared no potential conflicts of interest with respect to the research, authorship, and/or publication of this article.
